# Supplementary material for: Long-range population dynamics of anatomically defined neocortical networks
Source: eLife. 2016 May 24;5:e14679. doi: 10.7554/eLife.14679 (PMC4929001; doi:10.7554/eLife.14679)
Supplement: Figure 2—source data 1. — Table of optimum column size of each factor matrices related to neurons (N’ + N’offset), time points (T’), and trial conditions (C’) determined after cross-validation and cost function procedures for each animal used for denoising. Total possible column sizes are also indicated along with number of active neurons. DOI: http://dx.doi.org/10.7554/eLife.14679.008 [file elife-14679-fig2-data1.docx]

| **Animal ID** | **Active neurons** | $\boldsymbol{N}^{\mathbf{'}}\mathbf{+}\boldsymbol{N}_{\boldsymbol{offset}}^{\mathbf{'}}$  **(low / total )** | ***T’***  **(low / total)** | **C’**  **(low / total)** |
| --- | --- | --- | --- | --- |
| 1 | 23 | 30 / 283 | 6 / 40 | 5 / 6 |
| 2 | 80 | 73 / 330 | 15 / 40 | 6 / 6 |
| 3 | 55 | 26 / 274 | 6 / 40 | 4 / 6 |
| 4 | 29 | 12 / 301 | 3 / 40 | 4 / 6 |
| 5 | 120 | 51 / 267 | 11 / 40 | 5 / 6 |
| 6 | 150 | 72 / 350 | 14 / 40 | 6 / 6 |
| 7 | 35 | 18 / 190 | 4 / 40 | 4 / 4 |

**Figure 2 - Source data file 1. Optimized low tensor rank across animals.** Table of optimum column size of each factor matrices related to neurons (*N’* + *N’_offset_*), time points (*T’*), trial conditions (*C’*) determined after cross-validation and cost function procedures for each animal used for denoising. Total possible column sizes are also indicated along with number of active neurons.
